# A Clinical Manual of Diseases of the Ear

**Published:** 1872-02

**Authors:** 


*A Clinical Manual of Diseases of the Ear.* By Lawrence Turnbull, M. D., etc. Philadelphia, J. B. Lippincott, 1872.

This work is divided into twenty chapters and takes up in a regular and systematic order the anatomy of the ear and its functions. The Physiology of Hearing, Acoustics, Classification of Diseases of the Ear, Means of Diagnosis by use of Artificial and Daylight, Diseases and Injuries liable to occur to the Ear, and a Resume of the most effectual method of treating the more frequent affections of the ear.

We have not had sufficient time to give the book that careful attention which is so necessary in order to a complete review of the subject, but from what we have seen of it it impresses itself on us as a book destined to become one of the standard works of the day. The work is amply illustrated by over one hundred wood cuts and lithographs. On pages 416-419 is given a tabular view of the dissections of the ear in sixty-five deaf mutes, cases from Schwartze, Virchow and others which will be of interest to persons interested in the morbid anatomy of the part. The work concludes with a Bibliograph of the most important articles on the subject published from 1683 to 1871.

